# Individual gaze shapes diverging neural representations

**DOI:** 10.1073/pnas.2405602121

**Published:** 2024-08-30

**Authors:** Petra Borovska, Benjamin de Haas

**Affiliations:** ^a^Department of Experimental Psychology, Justus Liebig University, Giessen 35394, Germany; ^b^Center for Mind, Brain and Behavior, Marburg and Giessen, Darmstadt 35032, Germany

**Keywords:** individual differences, gaze behavior, complex visual stimuli, hyperalignment, fMRI

## Abstract

Complex visual stimuli evoke diverse patterns of gaze, but previous research suggests that their neural representations are shared across brains. Here, we used hyperalignment to compare visual responses between observers viewing identical stimuli. We find that individual eye movements enhance cortical visual responses but also lead to representational divergence. Pairwise differences in the spatial distribution of gaze and in semantic salience predict pairwise representational divergence in V1 and inferior temporal cortex, respectively. This suggests that individual gaze sculpts individual visual worlds.

Do individual brains represent the visual world in idiosyncratic ways? Is our view of complex visual stimuli unique? Given the foveal bias of the inferior temporal cortex (1, 2), ventral stream representations may be shaped by systematic idiosyncrasies of individual gaze (3) for static and dynamic stimuli (3–5). On the other hand, a range of previous results suggests that individual gaze may not matter much for the representation of complex, naturalistic stimuli: Eye-tracking studies have found higher interpersonal coherence for gaze toward directed content (6, 7) and stimuli with salient motion (8). Neuroimaging studies found that visually evoked brain activation in the ventral stream is only weakly modulated by eye movements (9) and may reflect broader visual field coverage (10) than previously assumed (1, 11, 12) and that individual representations of movie stimuli can be aligned across observers via linear transformations (13–16).

Here, we set out to test whether different individuals freely watching the same movie may nevertheless have individually divergent neural representations, which can be explained by idiosyncrasies in gaze. Specifically, we test whether the linear alignment of their neural representations is lower when observers freely watch a movie, as compared to fixating centrally while the movie is playing. Additionally, we test whether the degree of this representational divergence can be explained by systematic individual differences in gaze parameters for a given pair of observers.

## Results

**Fig. 1.**
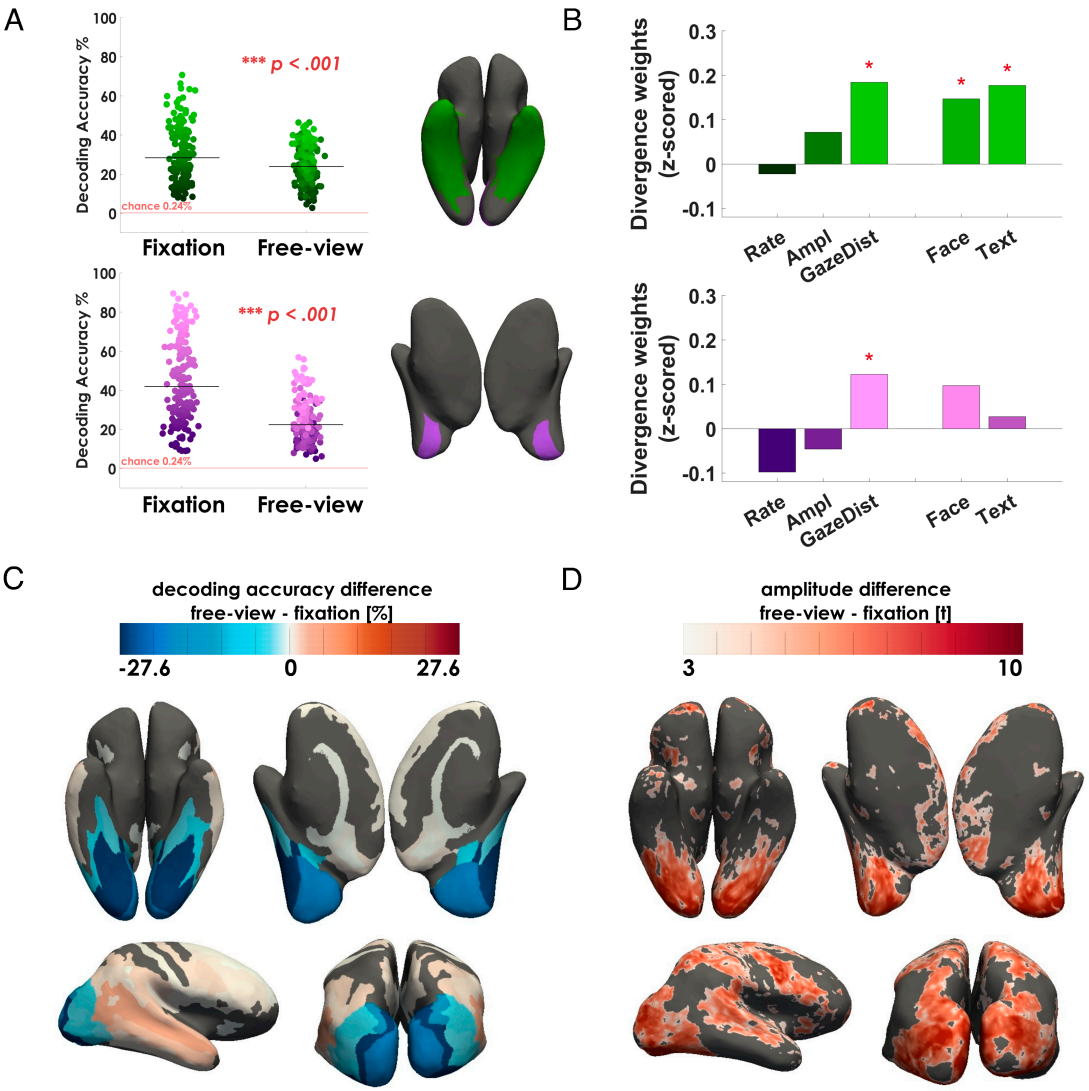
Results. (*A*) Cross-brain decoding accuracy in the fixation and free-viewing conditions for IT and V1. Each dot represents one pair of observers (*N* = 171 pairs), the chance level of 0.24 % is indicated by red lines, and *P*-values correspond to a GLMEs testing the effects of the conditions on cross-brain decoding accuracy. The *Top* (green) and *Bottom* (purple) plots show data from IT and V1 and the corresponding ROIs. The shades of dots correspond to decoding accuracy in the fixation condition. (*B*) Fitted weights of individual differences in low- and high-level gaze parameters predicting pairwise neural divergence in IT and V1. Simple main effects are shown for IT in green (*Top*) and V1 in purple (*Bottom*). Rate and Ampl: individual difference in the saccadic rate and amplitude; GazeDist: average Euclidean distance between gaze positions of two observers; Face and Text: individual difference in the tendency to fixate faces and text, respectively. Asterisks indicate significant simple main effects that survived Bonferroni correction. (*C*) Effects of free-viewing on neural alignment between observers. The heatmap shows the average difference in cross-brain decoding accuracy (in %) between free-viewing and fixation conditions across all pairs of observers on the inflated cortical surface. The color-coding indicates the magnitude of the difference for each region of interest (ROI), extracted using the Destrieux atlas parcellation and the Benson retinotopy atlas for V1, V2, and V3. Only differences that were significant at *P* < 0.001 according to the GLME are shown (*SI Appendix*, *Supporting Methods*). Values are color-coded as shown in the inset bar, with cool colors indicating a drop of decoding accuracy in the free-viewing condition. (*D*) Amplitude effects of free-viewing. The heatmap shows vertex-wise t-values for the contrast between free-viewing and fixation on the inflated cortical surface of an example observer. *t* values are color-coded as shown in the inset bar. Inflated hemispheres are shown in inferior (*Top Left*), medial (*Top Right*), lateral (*Bottom Left*), and posterior views (*Bottom Right*).

We let participants watch the same movie twice (*N* = 19, *SI Appendix*, *Supporting Methods* for sample details), once in an eye tracker and once in an fMRI session, the order of which was counterbalanced across participants. We tested effects of divergent gaze on neural representations, focusing on IT and V1 (Fig. 1*A*). Data from the scanning session were used for hyperalignment (15) to compare neural responses across observers: We fitted a linear transfer function to map the neural responses of one observer onto those of another and then applied it to holdout data of the same two participants and tested how well we could decode movie snippets. That is, we identified which movie snippet evoked a given response in an observer’s brain, based on predictions generated from responses in the other observer’s brain and the transfer function learned from independent data. Specifically, we used a Nearest Neighbor approach, identifying the highest correlating prediction across a library of predictions for hundreds of movie snippets. The accuracy of this cross-brain decoding was determined separately for each pair of observers and region of interest (ROI). It served as a proxy for the neural alignment of movie representations (*SI Appendix*, *Supporting Methods*).

To test the hypothesis that individual gaze leads to diverging neural representations, we tested two conditions in the fMRI session, instructing observers to either freely watch the movie or fixate centrally while it was playing. Cross-brain decoding dramatically decreased for free viewing compared to central fixation in V1 (24% vs. 45%, *b* = −0.2, *SE* = 0.01, *t*(682) = −15.83, *P* < 0.001; Fig. 1 *A*, *Bottom*) and IT (24% vs. 28%, *b* = −0.06, *SE* = 0.009, *t*(682) = 6.96, *P* < 0.001; Fig. 1 *A*, *Top*), as determined with a generalized linear mixed-effects model (*SI Appendix*, *Supporting Methods*). This shows that individual gaze leads to stronger but more idiosyncratic responses in V1 and IT, despite a significant amplitude increase of BOLD responses in V1 (*t*(18) = 3.53, *P* < 0.01), IT (*t*(18) = 4.32, *P* < 0.001), and beyond (Fig. 1*D*) for free-viewing compared to central fixation.

To further analyze the ways in which gaze shapes individual neural representations, we calculated pairwise differences for a range of gaze parameters during the eye-tracking session. We then tested the contribution of these pairwise differences in gaze to the pairwise cross-brain decoding accuracy in the free viewing condition in IT and V1 (Fig. 1*B*), while controlling for the corresponding accuracy in the fixation condition. To illustrate the contribution of differences in gaze to representational divergence, we flipped the sign of best fitting weights in this linear regression such that positive weights indicate a contribution to lower cross-brain accuracy during free-viewing.

Among low-level predictors, the pairwise Euclidean distance of gaze positions in the eye-tracking session significantly predicted representational divergence in the scanning session in both, V1 (*b* = 0.12, *SE* = 0.04, *t*(148) = 2.51, *P* < 0.05; Fig. 1 *B*, *Bottom*) and IT (*b* = 0.18, *SE* = 0.05, *t*(148) = 3.29, *P* < 0.01; Fig. 1 *B*, *Top*), but pairwise differences in saccadic amplitude and rate did not (all |*b*| ≤ 0.09, |*t*| ≤ 2.00, *P* ≥ 0.09; all *P*-values Bonferroni corrected, *SI Appendix*, *Supporting Methods*). Regarding semantic salience biases (3), pairwise differences in the tendency to fixate faces (*b* = 0.14, *SE* = 0.04, *t*(149) = 2.99, *P* < 0.01) and text (*b* = 0.17, *SE* = 0.05, *t*(149) = 3.48, *P* < 0.01; Fig. 1 *B*, *Top*) both significantly predicted pairwise representational divergence in IT, but not V1 (all |b| ≤ 0.09, |*t*| ≤ 2.02, *P* ≥ 0.08, all *P*-values Bonferroni corrected, *SI Appendix*, *Supporting Methods*).

Taken together, individual gaze led to enhanced but also more divergent neural responses in the early visual cortex and IT. The pairwise Euclidean distance of gaze positions during the eye-tracking session significantly predicted neural divergence in the later scanning session for both V1 and IT, whereas pairwise differences in the tendency to fixate faces and text significantly predicted neural divergence in IT only.

To explore the effects of divergent gaze more comprehensively, we expanded the comparison of cross-brain decoding accuracy between free viewing and central fixation to anatomical parcels encompassing the entire brain (*SI Appendix*, *Supporting Methods*). Results showed that the effect of neural divergence was largely confined to occipital and inferior temporal regions, with stronger effects in more posterior parts of IT and early visual areas (Fig. 1*C*).

## Discussion

Previous research has demonstrated that representations of complex visual stimuli in the human inferior temporal cortex (IT) can be decoded across brains using hyperalignment (13, 17). Here, we tested the hypothesis that such cross-brain decoding is limited by individual differences in gaze, leading to representational divergence.

Our findings show that free-viewing (compared to central fixation) leads to a strong increase of BOLD signal amplitudes across the visual system. This aligns with studies in humans and primates, showing enhanced neural responses to gaze shifts updating the visual input (7, 9, 18). Nevertheless, cross-brain decoding accuracy dramatically decreased in the free viewing condition, most prominently in the early visual cortex and posterior IT.

Moreover, pair-wise differences of gaze parameters in the eye-tracking session predicted the degree of gaze-induced representational divergence in the scanner. This was true for the average Euclidean distance between gaze positions in both V1 and IT, as well as for individual differences in the tendency to foveate faces and text in IT. This resonates with the foveal bias of face- and text-preferring neural populations in IT (1, 19, 20) and strongly suggests that individual gaze shapes individual visual worlds (3). Note that eye-tracking and fMRI sessions took place on different days and their order was counterbalanced across participants. Individual differences in gaze were highly consistent during the eye-tracking session, and previous studies have shown trait-like individual gaze biases (21, 22) which are shaped by genes (23, 24) and experience (25) and may be tuned to idiosyncratic characteristics of the visual system (26–28). Here, we find that individual gaze leads to systematic divergence of neural representations for identical complex visual stimuli. This matches recent results showing a relationship between individual differences in scene descriptions and gaze (29). Future studies should trace the developmental interplay between idiosyncrasies of the individual visual system, gaze, and perception. Furthermore, future research is needed to understand the consequences of representational divergence for communication, cooperation, individual preferences, and skills.

## Materials and Methods

Participants watched “Shaun the Sheep” in two sessions, once in an eye tracker (*N* = 39; *M*_age_ = 23.25; *SD* = 3.87; 27 females) and a subset of participants in an fMRI scanner (*N* = 19; *M*_age_ = 24.68; *SD* = 3.68; 12 females), with the order of experiments counterbalanced. Eye-tracking data were collected using an Eyelink 1000 (SR Research, Ottawa, Canada). The fMRI session used a 3-Tesla Siemens Prisma. Functional images were preprocessed with realignment, coregistration, smoothing, temporal filtering, and further denoising (*SI Appendix*, *Supporting Methods*). All participants provided written informed consent, and the study was approved by the local ethics committee of Justus-Liebig-University Giessen and in accord with the Declaration of Helsinki.

## Supplementary Material

Appendix 01 (PDF)

## Data Availability

Anonymized Eye-tracking and fMRI data have been deposited in Open Science Framework (https://osf.io/3pkw2/) (30).
