# Supplementary material for: Individual gaze shapes diverging neural representations
Source: Proc Natl Acad Sci U S A. 2024 Aug 30;121(36):e2405602121. doi: 10.1073/pnas.2405602121 (PMC11388360; doi:10.1073/pnas.2405602121)
Supplement: Supplementary file 1 — Appendix 01 (PDF) [file pnas.2405602121.sapp.pdf]

## Supporting Methods

### Participants

A total of 42 healthy participants with normal or corrected-to-normal vision took part in the eye-tracking experiment. The datasets of 3 participants were excluded (one due to excessive head movement, two because of missing datafiles), leaving a sample of 39 participants ($M_{age}$ = 23.25; *SD* = 3.87; 27 females). In a separate fMRI experiment, we recorded brain activity of 22 of these subjects. The dataset of 3 participants were excluded due to technical difficulties in the scanner, leaving a sample of 19 participants ($M_{age}$ = 24.68; *SD* = 3.68; 12 females). One additional participant was excluded from the analysis investigating the effect of gaze parameters on representational divergence, because of a recording failure in the eye-tracking session. All participants provided written informed consent, the study was approved by the local ethics committee of Justus-Liebig-University Giessen and in accord with the Declaration of Helsinki. Participants could choose between course credit or 10€/h for their participation.

### Design

Participants watched the audio-visual feature movie Shaun the Sheep in two separate sessions. The final sample of 19 participants saw the movie twice (on different days), once in the scanner and once in a separate session in an eye-tracker. The order of sessions was counter-balanced across participants (with 11 included participants in the eye-tracker first). An additional 20 participants watched the movie in the eye-tracker only.

### Eye-tracking experiment

#### *Apparatus*

Participants sat in a dark room with their head in a chin- and forehead-rest at a distance 55 cm from the screen. Gaze data were acquired using a tower mount Eyelink 1000 (SR Research, Ottawa, Canada) at a frequency of 1kHz. Stimuli were shown on a 23.8-inch LG Ultra HD monitor at a resolution of 3840 × 2160 pixels and a refresh rate of 59 Hz. Participants viewed the stimuli at a distance of ~55 cm and size of 48 x 27 degrees visual angle (dva). The experiment was programmed using Psychtoolbox version 3.0.16 (Kleiner et al., 2007) in MATLAB R2021b (Mathworks, Natick, MA, USA) on a Windows 10 PC.

### *Procedure*

Participants watched in the eye-tracker the first 50 minutes of the movie, divided into 10 blocks of 5 minutes each. Each block started with a calibration. Participants were instructed to freely view the movie, keep their head still and pay attention.

## Functional MRI experiment

### *Procedure*

The scanning session was divided into three runs, each lasting approximately ~21 mins. Each run consisted of four blocks presenting 5 mins of the movie. Participants were instructed at the beginning of each block to either fixate a super-imposed dot at the center of the movie or free-view for the following 5 mins. Each run had the same order of condition blocks: fixation, free-viewing, fixation, free-viewing. This sequence repeated for each run. At the end of each block, a blank screen was shown for 20s. In addition to these functional scans, we recorded an anatomical scan and field map for each participant.

### *Data acquisition*

The MRI session was carried out on a 3-Tesla imaging system (Siemens Prisma) with a 64-channel head coil at the Bender Institute of Neuroimaging (BION) at Giessen University. Stimuli were shown on a projector Epson EB-G5600 with a resolution of 1024 x 768 pixels and a refresh rate of 60 Hz. The stimulus video was rescaled to approximate the size of the stimulus in the Eyetracker with a width of ~48 dva and a height of ~27 dva. Eye movements were monitored online using a ViewPoint Eye-camera (Arrington Research Inc., Scottsdale, AZ) to ensure that participants followed the instructions in both conditions (fixating vs. free-viewing the movie).

Functional images covered the whole brain and were obtained using a multiband echo-planar imaging sequence (EPI) with an echo time (TE) of 33 ms and a repetition time (TR) of 1000 ms. Further parameters for obtaining functional data were as follows: Field of view (FoV) = 240 × 240 mm, in-plane resolution = 2.5 mm × 2.5 mm, 52 sagittal slices (descending) with a thickness of 2.5 mm and a distance factor of 20%, flip angle (FA) = 59°, acceleration factor = 4. Per participant, 3990 volumes of functional data were acquired (1330 per run).

High-resolution anatomical images were obtained using a T1-weighted magnetization-prepared rapid acquisition gradient-echo (MPRAGE) sequence with the following scan

parameters: FoV = 240 × 240 mm, TE = 3.53 ms, TR = 1880 ms, inversion time = 949 ms, in-plane resolution = 0.94 mm × 0.94 mm, number of slices = 176, slice thickness = 0.94 mm, flip angle (FA) = 8°.

Magnetic field perturbations were accounted for by measuring a field map with the following scan parameters: FoV = 220 × 220 mm, TE (1) = 10 ms, TE (2) = 12.46 ms, TR = 1,000 ms, in-plane resolution = 2.0 × 2.0 mm, slice thickness = 3.0 mm, number of slices = 40 (transversal), FA = 90°.

***Preprocessing***

All image files were converted to NIfTI format and preprocessed using SPM 12 (http://www.fil.ion.ucl.ac.uk/spm/software/spm12/) and custom MATLAB code. The remaining functional images were realigned and unwarped using the voxel displacement maps generated from the field maps. Further, the functional images were co-registered to the structural scan and spatially smoothed using a 4 mm Gaussian kernel[1]. Time series were bandpass filtered with a discrete cosine transform removing slow drifts and high frequency noise with a cut-off of 1/128 Hz, and subsequently z-scored. To remove noise, we extracted six rigid-body motion parameters and framewise displacements (all estimated during realignment), as well as three principal components from cerebrospinal fluid and white matter time-series. These nuisance regressors were regressed out from functional data for each run [2]. Finally, the first six volumes of all functional images were discarded to account for delays in the hemodynamic response.

## Data analysis

***Gaze consistency measures***

To quantify gaze parameters, we extracted saccades and fixations using the SR Research saccade detection algorithm (velocity >30 degrees/s and acceleration >8000 degrees/s2). Gaze coordinates that fell outside of video borders were excluded. Additionally, fixations with a duration under 100 ms were excluded (SR Research, 2022). This led to an exclusion of less than 1 % of fixations on average. To prevent erroneous gaze estimation during lid occlusion caused by a blink, saccades occurring 100 ms before or after a blink were also discarded (i.e., ~7 % of saccades on average were removed). Additionally, saccade and fixations with a duration > 1,000 or peak velocity > 1000 deg/s were removed.

We used several publicly available DNN algorithms to label the movie stimuli on a frame-wise basis. To label text, we used EAST (An Efficient and Accurate Scene Text Detector[3]). For face labels, we used YOLO5Face[4], and finally to label all remaining objects, we used YOLOV5[5]. We discarded all object-labels that were overlapping with text and face.

We included several gaze parameters for this and subsequent parts of the analysis. Fixations that fell within a distance of 0.5 dva from a given label, were marked accordingly, as face, text or other object fixations. We then calculated the proportion of text and face fixations among all labelled fixations for each observer. Similarly, we computed the median saccadic rate and amplitude for each observer. To assess the consistency of individual differences, we correlated individual gaze parameters across odd and even splits of the movie. Furthermore, gaze parameters served as basis for calculating pairwise differences used in linear regression models (see below, Effect of gaze parameters on cross-decoding accuracy).

We established that individual gaze varies in highly systematic ways, even for a directed movie (Shaun the Sheep). Individual differences in low-level parameters of viewing dynamics were large (variability up to factor 4) and highly consistent (split half consistency of saccadic rate $r(39) = .97$, $p < .001$; saccadic amplitude $r(39) = .94$, $p < .001$). Similarly, individual fixation biases towards faces and text varied up to factor 2 and showed moderate to good consistency (face $r(39) = .76$, $p < .001$; text $r(39) = .32$ , $p < .05$).

***Response amplitudes***

We specified boxcar regressors for blocks of fixation and free-viewing for each of the three runs and convolved them with the canonical hemodynamic response function, as implemented in SPM12. Each block had a duration of 5 mins. Additionally, we incorporated six motion parameters estimated during realignment. The conditions were contrasted against each other in a general linear model (free-view > fixation), generating *t*-maps for each participant (i.e. first level analysis). These *t*-maps were subsequently masked with the IT region of interest (ROI). ROI masks were individually defined for each hemisphere using the FreeSurfer (http://surfer.nmr.mgh.harvard.edu) parcellation algorithm [6] and included the following labels: G_oc-temp_lat-fusifor, G_oc-temp_med-Parahip, G_temporal_inf, Pole_temporal, S_collat_transv_ant, S_oc-temp_lat, S_oc-temp_med&Lingual, and S_temporal_inf. Finally, the masked *t*-maps were averaged for each participant and tested against zero using a one-sample *t*-test.

***Hyperalignment and cross-decoding accuracy***

For cross-brain decoding, we used a modified version of the hyperalignment technique[7,8]. Unlike classical hyperalignment, we directly fitted the data from one participant's cortical anatomy onto that of another, without projecting them into a common space. Hyperalignment uses Procrustes transformations to align the voxel spaces of individual subjects to one another based on their responses during movie watching[7]. We ran the classification algorithm separately for each condition and pair of observers. We calculated the cross-brain decoding accuracy in an a priori defined IT mask combining the labels listed above (see Response amplitudes) and in a mask for V1 (based on Benson retinotopy atlas). We used a similar approach to calculate cross-brain decoding accuracy in the whole brain by running our pipeline for each ROI separately. ROIs were extracted using the Freesurfer parcellation according to the Destrieux atlas and combined with the Benson retinotopy atlas for V1, V2, and V3[9–11]. This resulted in a total of 78 ROIs.

(1) In the first step (Learn) one member of the pair was assigned source and the other target. We divided the data for each condition into training and test sets of equal size. For voxel selection, we determined the highest correlation of each voxel in each ROI across its correlations with all voxels in the other brain's ROI. We then ranked voxels in each ROI based on this correlation score and selected the top 1000 voxels in both the target and source ROI for further analysis steps. Note that correlation scores and voxel selection were purely based on the training portion of data, but applied to the test data as well (cf. ref. [7]). Then, we used Procrustes transformation to find the best fitting linear transfer function from the training data of the source subject onto that of the target subject. (2) In the second step (Predict), we applied the resulting transformation matrix to the test data of the source subject to predict the test responses of the target subject. (3) In the last step (Evaluate), we divided the test data of the target subject and the corresponding predictions into 422 snippets of 18s each. Time snippets were derived using a sliding window with a step size of 2 s, and therefore partially overlapping. In each iteration the prediction snippets that overlapped with the target test snippet were discarded resulting in total of 422 test snippets in each iteration (cf. ref. [7]). Then, we determined the Nearest Neighbor prediction for each target test snippet as the prediction snippet correlating the highest with the target test snippet. If the Nearest Neighbor prediction corresponded to the same time-window of movie watching this was registered as a decoding success, otherwise as a failure. Decoding accuracy corresponds to the percentage of successes. Across both folds of training and test data chance level

corresponds to 0.24% (1/422 snippets). This procedure was repeated for all possible pairings of observers. Within each pair, decoding accuracy was averaged across both folds of training and test data and iterations in which either observer served as the target.

We performed a similar analysis for pre-defined IT and V1 ROIs, and extended our analysis to include all remaining ROIs extracted from the Destrieux and Benson atlases. Next, we tested for significant differences in cross-brain decoding accuracy between free-view and fixation conditions. Given the non-independence of the cross-brain decoding accuracy pairs ($N = 171$), we built Generalized linear mixed-effects models for each ROI to test an effect of conditions on cross-brain decoding accuracy. We included a categorical predictor indicating condition with two levels (Fixation and Free-view) and two random factors (Source and Target) expressing the identity of each subject, in which either observer served as the target. We confirmed that the cross-brain decoding accuracy significantly decreased for the free-view condition for several ROIs, while taking into account the identity of each subject. Additionally, we performed a conservative control analysis which reduced the degrees of freedom to the number of participants. For each observer, we averaged all pairwise instances of cross-brain decoding with this target observer. This is an index of how well this observer's activation patterns can be decoded on average, based on hyperalignment with all other brains. We did this separately for the free-viewing and central fixation conditions and entered the differences between conditions for each of our 19 observers into a one-sample *t*-test against zero, thus reducing degrees of freedom to 18. This conservative approach confirmed highly significant effects for both IT ($t(18) = 5.37$, $p < .001$) and V1 ($t(18) = 10.14$, $p < .001$).

Here, we list results of the first 12 ROIs with the largest decrease in cross-brain decoding accuracy in the free-view condition (which were all in early visual cortex and IT): V2 (51 % vs. 25 %, $b = -0.26$, $SE = 0.01$, $t(682) = -19.35$, $p < .001$); V3 (51 % vs. 24 %, $b = -0.26$, $SE = 0.01$, $t(682) = -20.24$, $p < .001$); V1 (45 % vs. 24 %, $b = -0.2$, $SE = 0.01$, $t(682) = -15.83$, $p < .001$); Ligual gyrus (50 % vs. 23 %, $b = -0.26$, $SE = 0.01$, $t(682) = -21.13$, $p < .001$); Occipital pole (37 % vs. 9 %, $b = -0.27$, $SE = 0.01$, $t(682) = -26.26$, $p < .001$); Superior occipital gyrus (34 % vs. 18 %, $b = -0.16$, $SE = 0.01$, $t(682) = -15.31$, $p < .001$); Middle occipital gyrus (27 % vs. 22 %, $b = -0.04$, $SE = 0.01$, $t(682) = -4.95$, $p < .001$); Posterior transverse collateral sulcus (25 % vs. 7 %, $b = -0.17$, $SE = 0.007$, $t(682) = -22.64$, $p < .001$); Calcarine sulcus (23 % vs. 16 %, $b = -0.07$, $SE = 0.007$, $t(682) = -9.2$, $p < .001$); Cuneus (21 % vs. 12 %, $b = -0.08$, $SE = 0.007$, $t(682) = -10.51$, $p < .001$); Lateral occipito-temporal gyrus (20 % vs. 13 %, $b = -0.06$, $SE = 0.007$, $t(682) = -8.57$, $p < .001$); and Inferior occipital gyrus and sulcus (19 %

vs. 12 %, $b = -0.07$, $SE = 0.007$, $t(682) = -10.33$, $p < .001$). All significant differences between conditions for all ROIs are displayed in the main figure (see Figure 1c).

To estimate the success of hyperalignment, we additionally conducted correlation based Nearest Neighbor classification on normalized data in IT, without using hyperalignment. First, we registered the functional data of each subject into standard MNI space using SPM12. Then, we repeated our classification pipeline (specified above) leaving out the Procrustes transformation to align brains of the pair of observers. This procedure resulted in a drastic drop of cross-brain decoding accuracy in both conditions (2.1 % in the fixation condition; 1.7 % in the free-view condition). We tested for the statistical significance of an effect of condition on the cross-decoding accuracy using GLME. This revealed a significant decrease in cross-brain decoding accuracy in the free-view compared to the fixation condition ($b = -0.003$, $SE = 0.0009$, $t(682) = -3.84$, $p < .001$).

***Effect of gaze parameters on cross-decoding accuracy***

To assess the impact of gaze parameters on representational divergence, we specified separate multiple linear regression models for IT and V1. Both models tested pairwise differences in gaze parameters during the eye-tracking session as predictors of pairwise representational divergence during the scanning session. Specifically, we regressed pairwise differences in gaze parameters onto pairwise cross-brain decoding accuracy in the free-viewing condition, controlling for the cross-brain decoding accuracy in the fixation condition. Then we flipped the sign of the resulting best fitting weights to display the contribution of diverging gaze to representational divergence (i.e. positive weights indicating a decrease in cross-brain decoding in the free-viewing condition). The first, low-level model included unsigned inter-observer differences in median saccadic amplitude and rate, as well as the median Euclidean distance of gaze positions as predictors. The second, high-level model, included unsigned individual differences in the proportion of labelled fixations falling onto faces and text. All predictors of interest except the Euclidean distance of gaze positions were normalized by their average across a given pair (to express differences relative to the overall magnitude of a given trait; for instance, the difference in saccadic rate between two observers was normalized by the average of the saccadic rate across these two observers). This was repeated for all pairs of observers, resulting in observer *dissimilarity* matrices for each gaze parameter and a corresponding matrix of pairwise decoding accuracies, each of which was vectorised and z-scored before being entered into the model. Resulting *p*-values were

Bonferroni corrected for three predictors in the low-level model and two predictors in the high-level model. models (two for each ROI).

Controlling for decoding accuracy in the fixation condition resulted in a significant effect on neural divergence in free-view condition for all models: low-level model in IT (*b* = 0.72, *SE* = 0.05, (148) = 13.002, *p* < .001); high-level model in IT (*b* = 0.72, *SE* = 0.05, *t*(149) = 14.32, *p* < .001); low-level model in V1 (*b* = 0.78, *SE* = 0.04, *t*(148) = 16.34, *p* < .001); high-level model in V1 (*b* = 0.79, *SE* = 0.04, *t*(149) = 16.35, *p* < .001).

1. Visconti di Oleggio Castello, M., Chauhan, V., Jiahui, G. & Gobbini, M. I. An fMRI dataset in response to “The Grand Budapest Hotel”, a socially-rich, naturalistic movie. *Sci Data* **7**, 1–9 (2020).

2. Zhou, X. *et al.* EAST: An efficient and accurate scene text detector. *Proceedings - 30th IEEE Conference on Computer Vision and Pattern Recognition, CVPR 2017* **2017-Janua**, 2642–2651 (2017).

3. Qi, D., Tan, W., Yao, Q. & Liu, J. YOLO5Face: Why Reinventing a Face Detector. *Lecture Notes in Computer Science (including subseries Lecture Notes in Artificial Intelligence and Lecture Notes in Bioinformatics)* **13805 LNCS**, 228–244 (2023).

4. Jocher, G. Ultralytics YOLOv5 (7.0). Preprint at https://doi.org/https://doi.org/10.5281/zenodo.3908559 (2020).

5. Destrieux, C., Fischl, B., Dale, A. & Halgren, E. Automatic parcellation of human cortical gyri and sulci using standard anatomical nomenclature. *Neuroimage* **53**, 1–15 (2010).

6. Haxby, J. V. *et al.* A common, high-dimensional model of the representational space in human ventral temporal cortex. *Neuron* **72**, 404–416 (2011).

7. Haxby, J. V., Guntupalli, J. S., Nastase, S. A. & Feilong, M. Hyperalignment: Modeling shared information encoded in idiosyncratic cortical topographies. *Elife* **9**, 1–26 (2020).

8. Benson, N. C., Butt, O. H., Brainard, D. H. & Aguirre, G. K. Correction of Distortion in Flattened Representations of the Cortical Surface Allows Prediction of V1-V3 Functional Organization from Anatomy. *PLoS Comput Biol* **10**, (2014).

9. Benson, N. C. *et al.* The retinotopic organization of striate cortex is well predicted by surface topology. *Current Biology* **22**, 2081–2085 (2012).

10. Avesani, P. *et al.* The open diffusion data derivatives, brain data upcycling via integrated publishing of derivatives and reproducible open cloud services. *Sci Data* **6**, 1–13 (2019).
